# Large Brunner's Gland Hyperplasia with Bleeding: A Case Report

**DOI:** 10.1155/2021/8861308

**Published:** 2021-06-11

**Authors:** Yuki Okutomi, Takaharu Kato, Hidetoshi Aizawa, Yuhei Endo, Naoya Kasahara, Fumiaki Watanabe, Hiroshi Noda, Toishiki Rikiyama

**Affiliations:** Department of Surgery, Saitama Medical Center, Jichi Medical University, 1-847, Amanuma-cho, Omiya-ku, Saitama 330-8503, Japan

## Abstract

We report a rare case of a large Brunner's gland hyperplasia (BGH) with severe anemia. A 33-year-old woman was transferred to our hospital with anemia and a duodenal mass. She had a 2-week history of melena and mild shortness of breath. Her hemoglobin level was 4.9 g/dl, and she required a blood transfusion. Abdominal computed tomography revealed a 7 cm tumor in the descending duodenum, and duodenoscopy revealed a polyp-like tumor with an ulcer at the duodenal bulb. We decided to perform surgery to prevent further bleeding. Intraoperatively, the tumor stalk was located at the anterior wall of the duodenal bulb; the ampulla was not involved, and we resected the tumor with the wall of the duodenal bulb. The resected tumor measured 7.0 × 4.0 × 2.3 cm, and pathologically, the tumor consisted of proliferated Brunner's glands in a small amount of fibrous stroma. The histological diagnosis was BGH with no malignancy. Most cases of BGH are benign and asymptomatic; however, it is important to be aware that some patients have severe anemia, gastrointestinal obstruction, or malignant potential.

## 1. Introduction

Brunner's glands are localized in the submucosal layer of the duodenum, and most are located in the proximal duodenum. Brunner's gland hyperplasia (BGH), also known as hamartoma, results from proliferation of Brunner's gland cells without malignancy. BGH is relatively rare, constituting less than 5% of benign duodenal tumors [1]. Owing to advances in esophagogastroduodenoscopy, diagnosing BGH has become more common, but the developmental mechanism is still unknown. The size and symptoms of BGH vary greatly, from asymptomatic to involving bleeding, and some cases have malignant tumors [2]. We report a rare case of a large BGH with severe anemia in a patient who required blood transfusion and surgical resection.

## 2. Case Report

A 33-year-old woman was transferred from a local hospital with anemia and a duodenal mass. She had a 2-week history of melena and mild shortness of breath before she consulted the local hospital. Examination revealed a hemoglobin concentration of 4.9 g/dl. Abdominal computed tomography (CT) revealed a 7 cm tumor in the descending duodenum, and duodenoscopy revealed a polyp-like tumor with an ulcer, at the duodenal bulb. Six units of red blood cells and 2 units of fresh-frozen plasma were given at that time, then she was transferred to our hospital.

**Figure 1:**
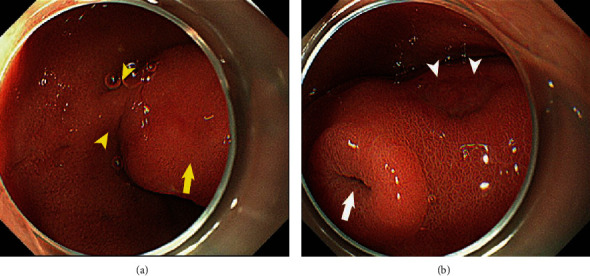
(a, b) Duodenoscopy showing a pedunculated (arrowheads in (a)), polypoid, 4 cm mass at the duodenal bulb. A secretory portion at the top of the tumor is visible (arrow in (b)) as well as an ulcerated lesion without bleeding (arrow in (a) and arrowheads in (b)).

**Figure 2:**
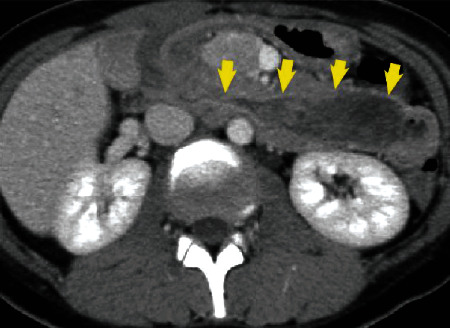
Abdominal CT showing a 7 cm mass extending from the bulb to the descending duodenum, with intussusception. The intrahepatic bile duct is not dilated.

**Figure 3:**
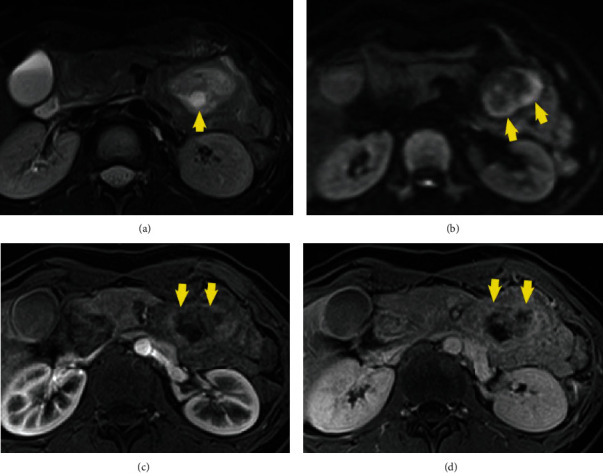
Magnetic resonance imaging (MRI) showing high- and low-signal intensities in the tumor on T2WI (a, b) and no enhancement during the arterial phase (c). The surface of the mass gradually enhanced during the delayed phase (d).

Physical examination on arrival revealed anemia and no abdominal pain. Her blood pressure, pulse rate, and respiratory rate were 114/63 mmHg, 96/min, and 18/min, respectively, and her hemoglobin concentration was 8.1 g/dl. Duodenoscopy revealed a pedunculated, polypoid 4 cm mass at the duodenal bulb. There was a secretory portion at the top of the tumor and an ulcerative lesion without bleeding (Figures 1(a) and 1(b)). Histopathology of the biopsy specimen revealed atypical inflammatory change without malignancy, and CT revealed a 7 cm mass extending from the bulb to the descending duodenum, with intussusception. The common bile duct was not dilated (Figure 2). Magnetic resonance imaging (MRI) showed combined high- and low-signal intensities in the tumor on T2-weighted image (T2WI). The tumor was not enhanced during the arterial phase; however, the tumor surface was gradually enhanced during the delayed phase (Figures 3(a)–3(d)).

**Figure 4:**
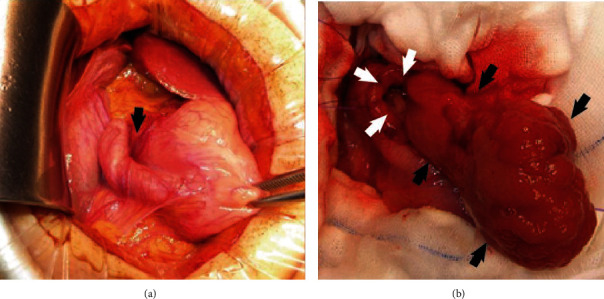
(a, b) The tumor stalk is located at the anterior wall of the bulb (a), and the ampulla is not involved. We opened the anterior duodenal wall (white arrows in (b)) and identified the tumor (black arrows in (b)). We resected the tumor with part of the wall of the duodenal bulb.

**Figure 5:**
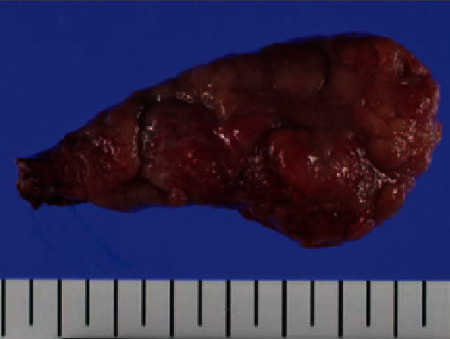
The resected tumor measured 7 × 4 × 2.3 cm. The surface epithelium consisted of normal duodenal mucosa with areas of focal ulceration and a secretory portion at the top.

**Figure 6:**
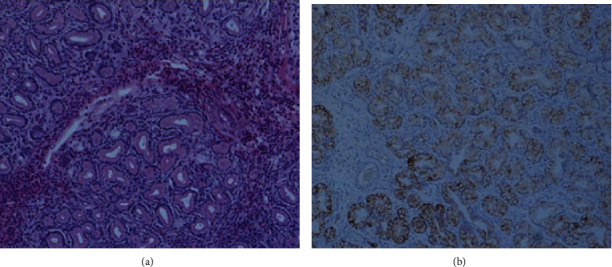
(a, b) The mass consists mostly of lobules of Brunner's glands showing no cellular atypia, with a small cluster of eosinophilic cells in the fibrotic lesion. Immunohistochemically, hyperplastic lobules of Brunner's glands were positive for Muc 6 (B).

We resected the duodenal tumor to prevent further bleeding and intestinal obstruction and to obtain a pathological diagnosis. Intraoperatively, the duodenal bulb was drawn into the descending duodenum, and the tumor stalk was located at the anterior wall of the duodenal bulb. The ampulla was not involved. We resected the tumor with the wall of the duodenal bulb (Figures 4(a) and 4(b)). The tumor had malignant potential as a gastrointestinal stromal tumor (GIST) or neuroendocrine tumor (NET), but we did not transition to enlarged resection because the patient was young, and the tumor was showing benign behavior. Macroscopically, the size of the resected tumor was 7 × 4 × 2.3 cm, the surface was smooth, and there were cystic lesions inside the mass (Figure 5). Microscopically, the tumor consisted of proliferated Brunner's glands in a small amount of fibrous stroma. The histological diagnosis was Brunner's gland hyperplasia (BGH) without malignancy (Figures 6(a) and 6(b)). The patient had an uneventful postoperative course and was discharged 7 days after the operation.

## 3. Discussion

We reported a patient who underwent surgery for BGH to prevent rebleeding and gastrointestinal obstruction. BGH is a submucosal tumor that must be distinguished from malignancies, such as GIST and NET. When considering BGH, because it consists of hyperplasia of secretory glands forming cysts, CT and MRI are useful to detect multiple cystic areas [3]. In our case, cystic lesions were not clearly formed, but there was a secretory portion at the top of the tumor and low signal intensity in the tumor on T2WI, suggesting that the tumor was secreting mucus. We considered the diagnosis as BGH at first, but could not deny the potential for malignancy, and the tumor size was large and bleeding; therefore, we chose surgical resection.

Clinical symptomatic presentation of BGH is caused by gastrointestinal bleeding or obstruction, leading to abdominal pain, nausea, vomiting, melena, or anemia. Two cases developed severe anemia and required blood transfusions [4, 5]. In some cases, when the tumor obstructed the ampulla of Vater, pancreatitis or jaundice occurred [6, 7].

Regarding the therapeutic strategy, BGH should be resected when the tumor size is >1 cm in diameter, which is more likely to be associated with symptoms, bleeding, and a higher risk of malignancy [8].

The present case had severe anemia and required blood transfusion, and we elected to perform surgery as quickly as possible. When there is a possibility of malignancy, as with a NET, patients must undergo surgery with lymph node dissection. In the current case, the biopsy specimen showed no signs of malignancy, and we performed simple tumor resection to prevent further bleeding, which resulted in avoiding pancreaticoduodenectomy.

In conclusion, most BGHs are benign tumors, but some cases have gastrointestinal obstruction, malignancy, and severe bleeding.
